# New signatures of poor CD4 cell recovery after suppressive antiretroviral therapy in HIV-1-infected individuals: involvement of miR-192, IL-6, sCD14 and miR-144

**DOI:** 10.1038/s41598-020-60073-8

**Published:** 2020-02-19

**Authors:** Francisco Hernández-Walias, María J. Ruiz-de-León, Isaac Rosado-Sánchez, Esther Vázquez, Manuel Leal, Santiago Moreno, Francesc Vidal, Julià Blanco, Yolanda M. Pacheco, Alejandro Vallejo

**Affiliations:** 1 Laboratory of Immunovirology, Department of Infectious Diseases. Health Research Institute Ramon y Cajal (IRyCIS), University Hospital Ramon y Cajal, Madrid, Spain; 2 Biomedicine Institute of Seville (IBiS), University Hospital Virgen del Rocío, Seville, Spain; 3 Department of Internal Medicine and Infectious Diseases. Hospital Viamed, Santa Ángela de la Cruz, Seville, Spain; 4 Infectious Diseases Unit and HIV/AIDS, Department of Internal Medicine, University Hospital Joan XXIII, IISPV, University Rovira i Virgili, Tarragona, Catalonia Spain; 5 AIDS Research Institute IrsiCaixa-HIVACAT, Research Institute of Health Sciences Germans Trias i Pujol, Badalona, Catalonia Spain; 6 University of Vic-University Central of Catalonia (UVIC-UCC), Vic, Catalonia Spain

**Keywords:** Biomarkers, Predictive markers

## Abstract

Up to 40% of newly diagnosed cases of HIV-1 infection are late diagnoses, with a profound decrease in CD4 cell counts in many cases. One-third of these individuals do not achieve optimal CD4 cell recovery (OR) after suppressive antiretroviral treatment (ART). This retrospective/longitudinal study of poor recovery (PR) included 79 HIV-1-infected individuals with CD4 count <200 cells/mm^3^ (25 PR and 54 OR) before ART. After suppressive ART, 21 PR and 24 OR individuals were further analysed, including paired samples. Selected miRs and plasma inflammatory markers were determined to investigate their potential predictive/diagnostic value for poor recovery. miR-192, IL-6 and sCD14 were independently associated with CD4 recovery before ART (p = 0.031, p = 0.007, and p = 0.008, respectively). The combination of these three factors returned a good discrimination (predictive value for PR) value of 0.841 (AUC, p < 0.001). After suppressive ART, miR-144 was independently associated with CD4 recovery (p = 0.017), showing a moderate discrimination value of 0.730 (AUC, p = 0.008) for PR. Our study provides new evidence on the relationship between miRs and HIV-1 infection that could help improve the management of individuals at HIV-1 diagnosis. These miRs and cytokines signature sets provide novel tools to predict CD4 cell recovery and its progression after ART.

## Introduction

Newly diagnosed cases of HIV-1 infection include individuals with a late diagnosis who often have a low level of CD4 cell counts. Antiretroviral treatment (ART) can restore the CD4 cell level in most of the HIV-1-infected individuals. Nevertheless, one-third of these individuals remained at a very low CD4 level (<200 cells/mm^3^) after ART despite virological suppression^1–3^.

Persistent immune activation and inflammation are associated with poor CD4 cell recovery (PR). These factors contribute to the risk of illness, increasing the risk of several morbidities compared to the uninfected population, as well as the risk of death^4,5^. While to date no effective alternative treatment is available to increase the CD4 cell levels to optimal counts, initiation of ART early after HIV-1 diagnosis might provide a good opportunity to maximise the CD4 cell recovery.

On the other hand, adding antiretroviral drugs to an already suppressive treatment does not improve either CD4 cell recovery nor reduce morbidity or mortality^6,7^. Besides, no observable clinical benefit was observed in IL-2 therapy, although it resulted in CD4 count increases^8^. The use of other immune-based therapies (e.g., growth hormones or IL-7) is controversial and its clinical benefit remains unclear^9^.

Micro RNAs (miRs) have been largely studied in cancer processes as biomarkers with an immunomodulatory role that might negatively or positively influence the immune system^10,11^. miRs are released inside exosome vesicles by cells and are present in all body fluids investigated to date. Disease presence and progression have been associated with an increase of both exosome release and their molecular content. These molecules could influence the homeostasis cell balance, promoting hematopoietic stem cells and, by modifying the levels of soluble cytokines, regulate the immune system^12–14^. A role for miRs in the pathogenesis of HIV-1 disease has been described^15,16^. The translation of HIV-1 proteins can be repressed by miRs located in resting CD4 cells contributing to the latency of HIV-1. On the other hand, HIV-1 itself can alter the expression of miRs expression influencing the progression of the disease^17–19^.

Since exosomes can modulate immune responses and might affect HIV-1 pathogenesis, we conducted this longitudinal study to quantify selected miRs and soluble inflammatory markers in HIV-1-infected individuals at ART onset and after 96 weeks under suppressive ART to investigate their potential predictive and diagnostic value of poor CD4 cell recovery.

## Methods

### Study setting and population

This retrospective/longitudinal study of adult HIV-1-infected individuals was performed with samples at ART onset and after 96 weeks of suppressive ART collected from the Spanish AIDS Research Network Cohort (CoRIS) through its HIV Biobank (Spain)^20,21^, and the HIV-1-infected individuals Cohort of the University Hospital Ramon y Cajal (Madrid, Spain). We selected 79 HIV-1-infected individuals with <200 CD4 cells/mm^3^ at ART onset who matched one of the following situations after 96 weeks under suppressive ART (<50 HIV-1 RNA copies/mL); (i) those whose CD4 count reached >250 cells/mm^3^ with cell increase >200 CD4 cells (OR, Optimal CD4 cell recovery individuals); and (ii) those whose CD4 count did not reach 200 cells/mm^3^ with cell increase <150 CD4 cells (PR, Poor CD4 cell recovery individuals). This very restrictive selection criterion allowed the comparison of two groups of individuals with no overlapping in CD4 cell increments that could result in confounding results.

Before ART initiation, 25 individuals with PR and 54 individuals with OR with available plasma samples were included in the study. For comparison, after 96 weeks of suppressive ART (the moment of categorisation into PR and OR), 21 individuals with PR and 24 OR were also analysed. Of them, 15 individuals with PR and 18 individuals with OR had paired samples (samples at ART onset and week 96 after treatment) to analyse the evolution of the biomarkers. Demographic parameters such as age, gender, and route of HIV-1 infection transmission, as well as the time of HIV-1 diagnosis, were collected from all individuals. The Ramón y Cajal Hospital Ethics Committee approved the study, which complied with the stipulations of the Declaration of Helsinki; all individuals gave their written informed consent to participate in this study.

### Laboratory measurements

CD4 and CD8 counts were determined in fresh blood with a FACScalibur flow cytometer (Becton Dickinson, Franklin Lanes, NJ, USA). Plasma HIV-1 RNA quantification was measured by quantitative polymerase chain reaction (qPCR, COBAS Ampliprep/COBAS Taqman HIV-1 test, Roche Molecular Systems, Basel, Switzerland) according to the manufacturer’s protocol, with a detection limit of 40 HIV-1 RNA copies/mL. HCV antibodies were assayed by EIA (Siemens Healthcare Diagnosis, Malvern, Pennsylvania), and plasma HCV RNA quantification by RT-qPCR (COBAS Amplicor, Roche Diagnosis, Barcelona, Spain).

### Plasma exosome-derived miR quantification

Briefly, frozen EDTA-plasmas were thawed and sequentially centrifuged to remove cell debris, as described previously^22^. They were then treated with thrombin and DNase to prevent platelet and DNA contamination, respectively, and filtered to eliminate larger vesicles such as large extracellular vesicles and apoptotic bodies. Exosomes were precipitated using the miRCURY Exosome isolation kit (Exiqon A/S, Vedbaek, Denmark), and quantified for exosome content by ExoELISA-ULTRA CD63 assay (SBI System Bioscience, Mountain View, CA, USA).

RNA from isolated exosome-enriched pellet was extracted using miRCURY RNA isolation kit-Biofluids (Exiqon A/S), and UniSp2-4-5 RNA templates added as an internal control. Concentration and purity of eluted RNA were analysed using a NanoDrop instrument (Thermo Scientific)^22^. All RNA samples with 260/280 ratio between 1.8 to 2 and a 260/230 ratio near 2 were considered suitable for further analysis. Ten nanograms of RNA was reverse transcribed in 15  µl reactions (mirCURY LNA Universal RT microRNA PCR, Exiqon A/S), including UniSp6 RNA spike-in template reaction control. cDNA was used for PCR reaction in triplicate using ExiLENT SYBR Green Master Mix (Exiqon A/S). LNA-based primers (Exicon A/S) for hsa-miR-451a and hsa-miR-23a were assayed to detect levels of haemolysis. Primers for hsa-miR-103a-3p, hsa-miR-425-5p, and hsa-miR-93-5p were quantified as reference controls for the calculation of the relative concentration of the following miRs: hsa-miR-106a-5p, hsa-miR-140-5p, has-miR-144-5p, hsa-miR-221-3p, hsa-miR-223-3p, hsa-miR-320a-5p, hsa-miR-409-5p, hsa-miR-192-5p, and hsa-miR-24-3p.

Thermocycler conditions were hot start at 95 °C/10 min followed by 40 cycles of 95 °C/15  s and 60 °C/45  s using LyghtCycler 480 II instrument (Roche, Basel, Switzerland). Amplification curves were analysed by Roche LightCycler 480 version 1.5.1.62 software. Reaction specificity was ascertained by performing the melt curve procedure. Ct values >35 and a standard deviation between triplicates >0.3 Ct, were considered unreliable and excluded from further analysis. The expression levels of single miRs relative to the mean reference miRs expression were calculated using the ΔCt method (Ct target miR minus mean references Ct)^23–25^ and reported as log_2_ 2^−ΔCt^.

### Soluble plasma biomarkers

Plasma samples were used to quantify the following markers of inflammation and immune activation: interleukin-6 (IL-6), soluble CD14 (sCD14), tumour necrosis factor-alpha (TNFα), IL-2, IL-17A, ICAM, and VCAM, using R&D Luminex HS assay (R&D Systems MN, USA). Human Procarta-Plex immunoassays (ThermoFisher Scientific) were used in combination with the Luminex instrument platform (MagPix, Luminex Corporation), following the manufacturer’s instructions.

### Statistical analysis

Continuous variables were expressed as the median and interquartile range (IQ_25–75_), and categorical variables by frequencies and proportions. The Mann-Whitney U test (non-parametric) for independent samples was used to compare continuous variables. Differences between categorical variables were evaluated using a contingency table (Chi-square distribution). For the categorical dependent variable (PR vs OR), univariate logistic regression analysis was assessed using all variables studied. Variables with p < 0.1 in the univariate analysis were included in the multivariate logistic regression analysis with a stepwise enter method. The regression coefficient (β) and 95% confidence interval for β were estimated in this model. Variables with p < 0.05 were independently associated with the dependent variable. The Wilcoxon signed-rank test was used to compare pared-samples to analyse the evolution of the biomarkers. Spearman’s rank correlation coefficient was used to measure the association between two variables. Besides, receiver operating characteristic (ROC) and area under the curve (AUC) were used to evaluate the diagnostic potential (with 95% confidence intervals), and to calculate the sensitivity and specificity of the biomarkers in CD4 cell recovery. Also, the likelihood ratio was performed to assess how these variables increase or decrease the probability to have poor CD4 recovery. Statistical analysis was performed using SPSS software 22.0 (SPSS Inc., Chicago, Illinois, USA).

## Results

The immunovirologic characteristics of the individuals at ART onset, classified subsequently as having either PR or OR, are shown in Table 1. The mean age of individuals was 40 years and were mainly males in both groups. No differences were found on the route of HIV-1 transmission, time from HIV-1 diagnosis to ART initiation, HIV-1 RNA load, and rates of HCV infection between the two groups of individuals. CD4 counts were lower in PR individuals, although this did not reach statistical significance (p = 0.076), while CD8 counts were similar in both groups. Nevertheless, the CD4/CD8 ratio was significantly lower in individuals with PR (p = 0.022).

**Table 1:** Immunovirologic characteristics of the patients at cART onset (predictive time point) and 96 weeks after cART (diagnosis time point).

	Poor CD4 T cell recovery individuals	Optimal CD4 T cell recovery individuals	p
N	25	54	
Age (years)	41 [35–52]	39 [34–48]	0.340
Gender (males, %)	21 (84%)	46 (85.2%)	0.875
Route of HIV-1 transmission*			0.287
Injecting drug use (N, %)	4 (16%)	10 (18.5%)	
Sexual intercourse (N, %)	16 (64%)	38 (70.4%)	
Unknown	5 (20%)	6 (11.1%)	
HIV-1 diagnosis to ART initiation (months)	1.0 [0.2–2.0]	2.0 [1.0–26.0)	0.147
Type of ART initiated*			0.252
2 NRTIs + 1 PI	14 (56%)	23 (42.6%)	
2 NRTIs + 1 NNRTI	9 (36%)	26 (48.1%)	
2 NRTIs + 1 INSTI	1 (4%)	3 (5.6%)	
3 NRTIs	1 (4%)	2 (3.7%)	
HIV-1 RNA load (log_10_ copies/mL)	5.01 [4.23–5.33]	5.10 [4.69–5.51]	0.197
HCV infection*			0.349
Positive for anti-HCV antibodies	5 (20%)	14 (25.9%)	
HCV PCR positive	3	12	
HCV PCR unknown	2	2	
Negative for anti-HCV antibodies	8 (32%)	20 (37%)	
Unknown for anti-HCV antibodies	12 (48%)	20 (37%)	
CD4 T cell count (cells/mm^3^)	84 [57–146]	139 [67–171]	0.076
CD8 T cell count (cells/mm^3^)	645 [464–831]	678 [434–912]	0.804
CD4/CD8 ratio	0.11 [0.07–0.23]	0.20 [0.14–0.27]	0.022
96 weeks after cART onset			
CD4 T cell count (cells/mm^3^)	192 [136–214]	429 [362–563]	<0.001
CD8 T cell count (cells/mm^3^)	553 [388–1066]	867 [692–1109]	0.023
CD4/CD8 ratio	0.26 [0.17–0.46]	0.53 [0.39–0.70]	0.001
CD4 T cell increment	77 [54–124]	318 [248–437]	<0.001

Median and interquartile range (IQ25–75) for continuous variables, and frequencies and proportions for categorical values. ART, antiretroviral treatment. NRTI,nucleoside/nucleotide reverse transcriptase inhibitor; NNRTI, non-nucleoside reverse transcriptase inhibitor; PI, protese inhibitor; INSTI, integrase strand transfer inhibitor, Mann Whitney U test for continuous values and * Fisher’s exact test (two tailed) for categorical values. Significant when p < 0.05 in bold.

The type of antiretroviral treatment prescribed was similar in both groups (p = 0.252), as shown in Table 1. Most of the PR individuals (56%) received two nucleoside/nucleotide reverse transcriptase inhibitors (NRTIs) plus one protease inhibitor (PI). Emtricitabine plus tenofovir were the most prescribed NRTI combination (47.8%), while the most prescribed PIs were ABT (38.8%) and atazanavir potentiated with ritonavir (17.4%). Nine PR individuals (36%) received two NRTIs plus one non-nucleoside reverse transcriptase inhibitor (NNRTI), with emtricitabine plus tenofovir as the most prescribed combination of NRTIs (80.7%), and efavirenz as the most prescribed NNRTI (92.3%). Among the OP individuals who received two NRTIs plus one PI (42.6%), emtricitabine plus tenofovir was again the most prescribed combination of NRTI (78.6%), while either lopinavir, ABT, or darunavir englobed the 78.5% of the PIs. Twenty-six OP individuals (48.1%) received two NRTIs plus one NNRTI, with emtricitabine plus tenofovir as the most prescribed combination of NRTIs (55.6%), and either nevirapine (44.4%) or efavirenz (55.6%) as the prescribed NNRTI.

After 96 weeks under suppressive ART (mostly composed by two nucleoside analogue inhibitor plus one protease inhibitor), CD4 counts, CD8 counts, and CD4/CD8 ratio were significantly lower in individuals with PR (p < 0.001, p = 0.023, and p = 0.001, respectively) (Table 1). Also, the CD4 cell increase was lower in individuals with PR (p < 0.001) as a consequence of the group definition criteria.

### Exosome-derived miRs and soluble cytokines with predictive value for CD4 cell recovery at ART onset

miR-106a and miR-140 levels were lower in individuals with PR (p = 0.001 and p = 0.002, respectively), while the miR-192 level was higher (p = 0.001) compared to individuals with OR (Table 2A). Besides, similar levels of the rest of the miRs were found in the two groups of individuals. Plasma IL-2 levels were lower in individuals with PR compared to individuals with OR (p = 0.017), while the levels of IL-6 and sCD14 were higher (p < 0.001 and p = 0.046, respectively) (Table 2B). Also, individuals with PR showed higher VCAM and ICAM levels compared to those with OR, although not reaching the limit of statistical significance (p = 0.051 and p = 0.055, respectively). The levels of the rest of the soluble biomarkers were similar between the two groups of individuals.

**Table 2:** miRNAs and cytokines levels of the patients at cART onset.

	Poor CD4 T cell recovery patients N = 25	Optimal CD4 T cell recovery patients N = 54	p
**A. miRs**			
miR-106a	−1.36 [−2.52–−0.27]	0.09 [−0.98–1.36]	**0.001**
miR-140	−1.47 [−2.54–−0.35]	−0.36 [−1.26–0.64]	**0.002**
miR-144	−1.79 [−4.18–−0.37]	−3.41 [−4.62- −1.26]	0.219
miR-221	−1.08 [−2.19–0.16]	−0.75 [−1.61–0.40]	0.141
miR-223	2.87 [1.59–4.24]	2.97 [1.39–4.89]	0.891
miR-320a	1.66 [0.72–2.54]	1.63 [0.53–2.92]	0.929
miR-409	−2.96 [−4.18–−0.49]	−3.69 [−4.66–−0.49]	0.212
miR-192	0.70 [−0.91–2.18]	−1.24 [−1.74–0.03]	**0.001**
miR-24	2.04 [0.38–3.02]	1.84 [0.13–3.57]	0.887
**B. Cytokines**			
IL-2 (pg/ml)	0.71 [0.57–0.99]	0.98 [0.64–1.35]	**0.017**
IL-6 (ng/mL)	8.29 [5.53–10.95]	4.52 [2.89–6.49]	**<0.001**
IL-17A (pg/mL)	0.69 [0.45–0.99]	0.77 [0.59–1.06]	0.244
TNF-α (pg/mL)	4.12 [2.44–5.32]	2.55 [1.45–4.33]	0.056
sCD14 (ng/ml)	3.19 [2.51–4.01]	2.89 [1.76–3.40]	**0.046**
ICAM (log_10_ ng/mL)	3.74 [3.01–4.76]	3.38 [2.44–4.14]	0.051
VCAM (log_10_ ng/mL)	1.12 [0.80–1.45]	0.90 [0.62–1.22]	0.055

Median and interquartile range (IQ25–75). Mann Whitney U test. Significant when p < 0.05 in bold.

To investigate whether gender or HCV infection could have had any effect on the levels of the variables studied, only individuals with OR were analysed since the number of individuals with PR (only four females and three HCV-infected individuals) was not enough to give good statistical power in the comparison. Hence, among individuals with OR no statistical differences in the levels of the miRs and cytokines were found between females (n = 8) and males (n = 46) (Supplementary Tables S1A,B). Regardless that the diagnosis of HCV infection was not documented in a large number of individuals, an analysis of the impact of HCV infection among individuals with OR was performed including 12 individuals with OR with positive anti-HCV antibodies and positive HCV PCR, and 20 individuals with OR with negative anti-HCV antibodies. Individuals with unknown HCV PCR were not included in the analysis because there could be cases of spontaneous HCV clearance that would interfere with the results. Hence, no significant differences were found in the levels of miRs between individuals with or without HCV infection, except for miR-192 that was shown to be higher in individuals with PR (p = 0.010) (Supplementary Table S1A).

### Exosome-derived miR-192, IL-6, and sCD14 were independently associated with CD4 cell recovery at ART onset

To identify which biomarkers were independently associated with CD4 cell recovery, multivariate logistic regression analysis (PR vs OR) was performed including all variables with p < 0.1 in the univariate logistic regression test (Table 3). Hence, miR-192, IL-6, and sCD14 were independently associated to CD4 cell recovery (p = 0.031, p = 0.007, and p = 0.008, respectively). In addition, while no correlation between these three biomarkers was found, miR-192 correlated directly with TNF-α (r = 0.524, p = 0.014) and ICAM (r = 0.536, p = 0.010), and inversely with IL17A (r = −0.458, p = 0.063). Besides, miR-106a correlated inversely with IL-6 (r = 0.479, p = 0.041), while miR-140 correlated directly with IL-2 (r = 0.477, p = 0.45) and inversely with sCD14 (r = 0.481, p = 0.039).

**Table 3:** Uni and multivariate analysis to investigate which variable was independently associated with CD4 T cell recovery (dependent variable poor vs. optimal CD4 T cell recovery).

	CD4 T cell recovery	
	Univariate logistic regression p, β coefficient (95% CI)	Multivariate logistic regression p, β coefficient (95% CI)
CD4 T cell count	0.083, 0.993 (0.985–1.001)	
CD4/CD8 ratio	0.035, 0.001 (0.000–0.589)	
miR-106a	0.001, 0.568 (0.402–0.802)	
miR-140	0.001, 0.465 (0.292–0.741)	
miR-221	0.096, 0.755 (0.542–1.052)	
miR-192	0.002, 1.609 (1.198–2.162)	**0.031**, 1.725 (1.052–2.830)
IL-2	0.015, 0.178 (0.045–0.712)	
IL-6	0.001, 1.290 (1.104–1.507)	**0.007**, 1.497 (1.114–2.011)
sCD14	0.003, 2.413 (1.345–4.330)	**0.008**, 5.276 (1.554–17.915)
ICAM	0.063, 1.479 (0.980–2.234)	
VCAM	0.085, 2.769 (0.867–8.838)	

Only variables with p < 0.1 in the univariate logistic regression were analysed in the multivariate logistic regression analysis. CI: confidence interval. Significant when p < 0.05.

**Figure 1:**
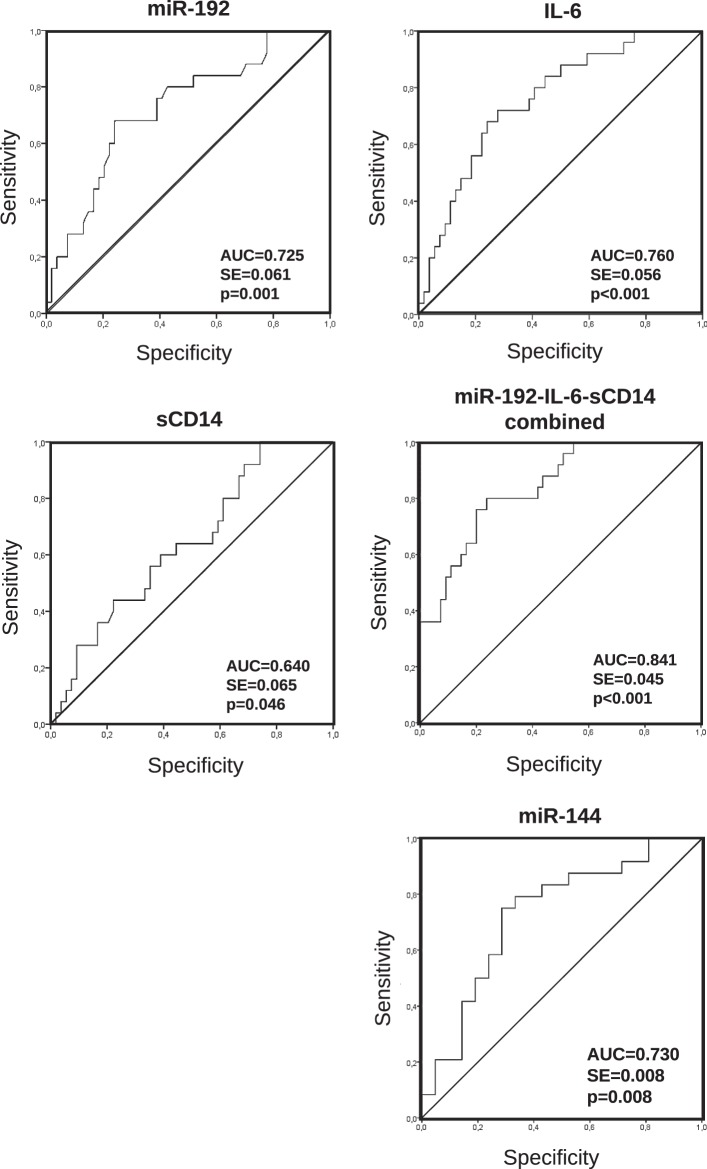
Receive operating characteristic (ROC) curves of differentially expressed miR-192, IL-6, sCD14 and the combination of these three factors between individuals with poor and optimal CD4 T cell recovery at ART onset, and miR-144 after 96 weeks of suppressive ART. AUC, area under the curve; SE, standard error.

ROC curves of miR-192, IL-6 and sCD14 showed moderate discrimination with an AUC value of 0.725 (95% CI: 0.605–0.845), 0.760 (95% CI: 0.650–0.870), and 0.640 (95% CI: 0.514–0.767), respectively (Fig. 1). Nevertheless, the combination of these three factors showed stronger discrimination with an AUC value of 0.841 (95% CI 0.752–0.929). The sensitivity and specificity of miR-192, IL-6, sCD14, and the combination of these three factors to predict PR with an optimal cut-off value are summarised in Supplementary Table S2. The combination of these factors increases by 3.16-times the probability to predict poor CD4 cell recovery (likelihood ratio analysis). No correlation between miR-192 and CD4 count was found (data not shown), while IL-6 and sCD14 negatively correlated with CD4 count (p = 0.004 and p < 0.001, respectively).

### Levels of exosome-derived miRs and soluble cytokines after 96 weeks of suppressive ART

Twenty-one individuals with PR (15 individuals with samples at ART onset) and 24 individuals with OR (18 individuals with samples at ART onset) were analysed after 96 weeks of suppressive ART (Supplementary Table S3). As expected, the CD4 count and CD4/CD8 ratio were lower in individuals with PR (p < 0.001 and p = 0.002, respectively), while the CD8 count was similar in both groups.

After suppressive ART, miR-106a, miR-140, and miR-144 showed lower levels in individuals with PR (p = 0.003, p = 0.048 and p = 0.008, respectively) (Table 4A). IL-2 levels were lower in individuals with PR (p = 0.014), while the levels of IL-6 and sCD14 were higher in those individuals (p = 0.005 and p = 0.002, respectively) (Table 4B).

**Table 4:** Post suppressive cART levels (at week 96) of miRs and cytokines of the patients.

	Poor CD4 T cell recovery patients N = 21	Optimal CD4T cell recovery patients N = 24	p
**A. miRs**			
miR-106a	−0.25 [−5.31–2.28]	2.74 [0.81–3.60]	**0.003**
miR-140	0.89 [−5.30–1.75]	1.71 [−2.52–3.00]	**0.048**
miR-144	−3.93 [−5.22–2.46]	−2.44 [−2.93–1.50]	**0.008**
miR-221	−1.34 [−4.10–0.61]	−0.78 [−2.46–0.31]	0.045
miR-223	3.49 [2.25–4.39]	3.81 [2.79–4.62]	0.317
miR-320a	1.72 [0.23–2.32]	1.74 [1.00–2.42]	0.601
miR-409	−1.25 [−3.17–0.45]	−1.32 [−2.01–0.19]	0.873
miR-192	−0.89 [−1.87–0.19]	−1.20 [−2.12–0.08]	0.577
miR-24	−0.30 [−1.26–1.92]	0.35 [−1.00–1.53]	0.419
**B. Cytokines**			
IL-2 (pg/ml)	0.61 [0.41–0.85]	0.97 [0.69–1.14]	**0.014**
IL-6 (ng/mL)	3.82 [3.27–5.16]	2.41 [1.37–4.27]	**0.005**
IL-17A (pg/mL)	1.29 [0.92–1.54]	1.48 [1.17–1.83]	0.052
TNF-α (pg/mL)	2.64 [1.61–4.13]	1.97 [1.24–2.79]	0.162
sCD14 (µg/ml)	2.44 [2.12–2.94]	1.89 [1.51–2.36]	**0.002**
ICAM (log_10_ ng/mL)	2.05 [1.53–2.92]	1.66 [1.34–2.31]	0.069
VCAM (log_10_ ng/mL)	0.79 [0.58–0.96]	0.59 [0.50–0.87]	0.097

Median and interquartile range (IQ25–75). Mann Whitney U test. Significant when p < 0.05 in bold.

### Exosome-derived miR-144 was independently associated with CD4 cell recovery after 96 weeks of suppressive ART

After performing multivariate logistic regression analysis with all variables with p < 0.1 in the univariate logistic regression test, only miR-144 was independently associated with CD4 cell recovery (p = 0.017) after 96 weeks of suppressive ART (Table 5). Also, the ROC curve of miR-144 showed moderate discrimination with an AUC value of 0.730 (95% CI: 0.579–0.882) (Fig. 1). The sensitivity and specificity of miR-144 to diagnose PR with an optimal cut-off value are summarised in Supplementary Table S2. This miR increased by 2.16-times the probability to diagnose poor CD4 recovery. Of note, miR-144 positively correlated with CD4/CD8 ratio (p = 0.006) and almost correlated with CD4 count (p = 0.082).

**Table 5:** Uni and multivariate analysis to assess which variable was independently associated with CD4 T cell recovery (dependent variable poor vs. optimal CD4 T cell recovery) after 96 weeks of suppressive cART.

	CD4 T cell recovery (poor vs. optimal recovery)	
	Univariate logistic regression P, β coefficient (95% CI)	Multivariate logistic regression p, β coefficient (95% CI)
CD4/CD8 ratio	0.023; 0.000 (0.000–0.277)	
miR-106a	0.003; 0.688 (0.538–0.881)	
miR-140	0.098; 0.852 (0.704–1.030)	
miR-144	0.017; 0.618 (0.417–0.916)	**0.017**, 0.089 (0.012–0.654)
miR-221	0.039; 0.680 (0.471–0.981)	
IL-2	0.027; 0.114 (0.017–0.785)	
IL-6	0.010; 1.769 (1.145–2.734)	
IL-17A	0.053; 0.201 (0.040–1.019)	
sCD14	0.006; 5.564 (1.639–18.884)	
ICAM	0.074; 1.953 (0.936–4.072)	

Only variables with p < 0.1 in the univariate logistic regression were analysed in the multivariate logistic regression analysis. CI: confidence interval. Significant when p < 0.05.

### Evolution of the levels of exosome-derived miRs and soluble cytokines after 96 weeks of suppressive ART

**Figure 2:**
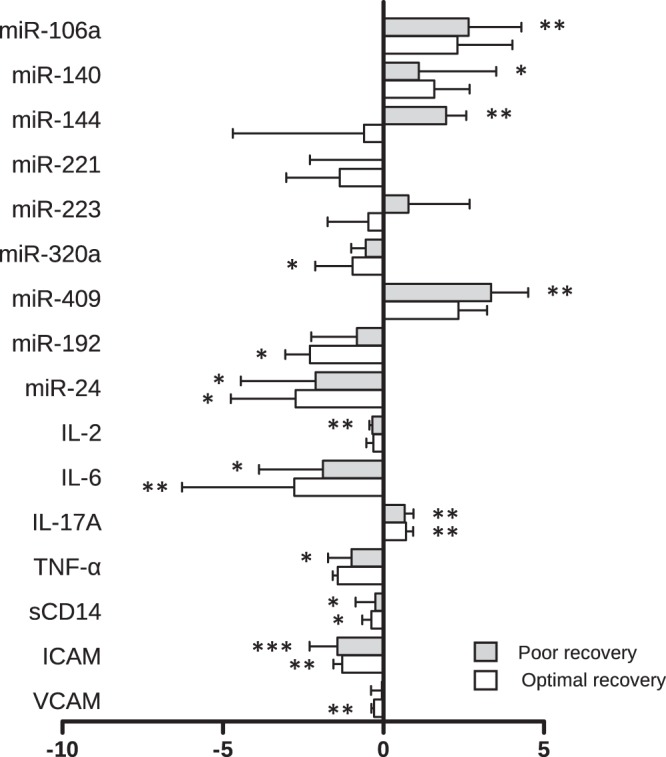
Increments of the levels of miRs and cytokines from ART onset to after 96 weeks of suppressive ART. *p < 0.05; **p < 0.01 and ***p < 0.001.

Individuals with paired samples were analysed to investigate the evolution of the levels of the biomarkers between these two groups during suppressive ART. Hence, the levels of miR-106a, miR-140, miR-144, and miR-409 significantly increased only in individuals with OR (p = 0.001, p = 0.014, p = 0.008, and p = 0.006, respectively), while similar levels of these miRs were found in individuals with PR (Fig. 2). In contrast, the levels of miR-320a and miR-192 decreased significantly only in individuals with PR (p = 0.012 and p = 0.002, respectively). Also, miR-24 significantly decreased in both groups of individuals (p = 0.004 and p = 0.012, respectively).

The levels of IL-2 and TNF-α significantly decreased in individuals with OR (p = 0.010 and p = 0.011, respectively), while the level of VCAM decreased only in individuals with PR (p = 0.002). On the other hand, the levels of IL-6, sCD14, and ICAM significantly decreased in both groups of individuals, while the level of IL17A significantly increased in both groups of individuals. Both miR-106a and miR-140 correlated directly with IL2 (r = 0.586, p = 0.021, and r = 0.595, p = 0.017, respectively), while miR-192 correlated inversely with IL17A (r = 0.586, p = 0.021). No correlation of miR-144 with inflammation markers was found.

## Discussion

To find predictive biomarkers at ART onset to identify earlier those HIV-1-infected individuals who will have poor CD4 cell recovery after suppressive ART is crucial for the good management of HIV-1-infected individuals^4,26^. This study showed that, at ART onset, lower expression of miR-106a and miR-140, and higher expression of miR-192 could be found in individuals who, thereafter, will be classified as having poor CD4 cell recovery. In parallel, higher levels of IL-6 and sCD14 and lower levels of IL-2 were found in these individuals. These results provide these biomarkers with a remarkable value that deserves to be further analysed. Interestingly, multivariate analysis showed that miR-192, IL-6, and sCD14 biomarkers were independently associated with CD4 cell recovery at ART onset, and could help strongly identify individuals with subsequent poor CD4 cell recovery after suppressive ART. These differences were independent of both gender and HCV infection (tested among individuals with optimal CD4 cell recovery) except for miR-192, whose level was significantly lower in individuals with HCV infection. This shows that HCV infection could modify the expression of some miRs. Also, the combination of miR-192, IL-6, and sCD14 provided good predictive value with a slight increase (by 3.16-times) in the probability to predict poor CD4 cell recovery.

Little is known regarding miRs profiles in HIV-1-infected individuals with poor CD4 cell recovery before ART onset. Instead, some recent works have been focused on different scenarios, including differential profiles compared to uninfected individuals, the effect of ART among HIV-1-infected individuals, and analysing HIV-1 controllers^22,27–29^. It is important to investigate the dynamic changes in human regulation of miRs during HIV-1 infection since they can regulate viral replication and translation and might play an important role in HIV-1 pathogenesis and latency^17,30,31^. The interaction of HIV-1 with cellular miRs expressed in infected cells has been controversial. Many groups have reported that cellular miRs bind to HIV and reduce viral gene expression by interfering virion infectivity and virion miR function, facilitating in some degree the HIV-1 latency^30–32^. On the contrary, a few reports have shown that miRNAs bind HIV-1 transcripts very inefficiently^19,33^. However, these last two studies (from the same laboratory) were restricted to early time points after HIV-1 infection, which is a different setting from the present study in which the individuals had a long course of infection.

Although ongoing inflammation (including sCD14 and IL-6) has been previously associated with HIV-1 disease progression, some authors have reported that the baseline sCD14 level was positively associated with CD4 cell recovery^34,35^. These authors suggested a potential explanation where a high level of sCD14 might be protective against LPS-induced immune activation due to a high ratio of sCD14 compared to LPS-binding protein. In contrast, we had previously shown that higher sCD14 level was associated with an increase in markers of HIV-1 progression after suppressive ART^36^, and was found in individuals with low CD4 count after suppressive ART^37^, although no data before ART were available. Besides, we also reported increased levels of IL-6 in individuals with low CD4 cell recovery before ART onset^38^.

In line with our results, miR-192 has been reported to inhibit cell proliferation and induce apoptosis *in vitro*^39,40^ and rheumatoid arthritis^41^. Also, p53 can induce miR-192 to promote its activation facilitating cell cycle arrest^42,43^. Besides, downregulation of miR-140 has been correlated with higher levels of inflammatory factors *in vitro*^44^ and its upregulation correlated to low levels of IL-6 among other inflammation cytokines^45^. On the other hand, miR-106a has been reported to prevent Th17-mediated inflammation^46,47^. Of note, this study showed that miR-192 correlated directly with TNF-α and inversely with IL17A, while miR-106a correlated inversely with IL-6 and miR-140 correlated directly with sCD14. On the contrary, miR-106a did not correlate with IL17A in this study perhaps due to the relatively small number of studied individuals. These events are in agreement with our results, where upregulation of miR-192 and, to a lesser extent, downregulation of miR-140 and miR-106a could promote the inhibition of cell proliferation, induction of apoptosis, and increase in inflammatory biomarkers, facilitating the impairment for CD4 recovery in individuals with future poor CD4 cell count recovery under ART.

After 96 weeks of suppressive ART, when individuals were categorised as having either poor CD4 cell recovery or optimal CD4 cell recovery, the expression of miR-106a and miR-140 were still lower in individuals with poor CD4 cell recovery, with a significant increase only in individuals with optimal CD4 cell recovery. Also, the expression of miR-192, although higher compared to individuals with optimal CD4 cell recovery, did not reach statistical significance. Of note, while miR-144 was downregulated compared to individuals with optimal CD4 cell recovery, its levels increased in individuals with optimal CD4 cell recovery and decreased in individuals with poor CD4 cell recovery. This miR has been reported to inhibit inflammatory cytokines like TNF-α and IFN-γ during active tuberculosis^48^, which is in line with that found in this study, where the downregulation of miR-144, in combination with the downregulation of miR-106a and miR-140, could facilitate the expression of inflammatory cytokines. Both miR-106a and miR-140 correlated directly with IL-2, which is a multifunctional cytokine with potent activity as a T cell growth factor. Although miR-144 did not correlate with inflammation biomarkers, in contrast to elsewhere reported^48^, miR-192 still correlated inversely with IL-17A, promoting IL17-mediated inflammation.

In parallel, analysing paired samples, the levels of IL-6, and sCD14 decreased during treatment and remained higher in individuals with poor CD4 cell recovery. Besides, the level of IL-2 was lower in individuals with poor CD4 cell recovery, similar to those found at ART onset. After multivariate analysis, only miR-144 has independently associated with CD4 cell recovery with moderate AUC and a slight increase of the probability to be classified to the poor CD4 cell recovery profile.

Limitations of this study included the lack of stored peripheral mononuclear cells to perform cellular immunology analysis, the relatively small number of individuals, and the limited number of paired samples available to analyse the evolution of the biomarkers. Also, there are differences between the groups that could alter the results, such as other viral infections. Besides, the methodology used for this study does not reveal molecular mechanisms about the causes of miRs deregulation. Whether these miRs are actively involved in the progression of HIV-1 infection or are markers for disease pathogenesis remains unclear. On the other hand, the strength of this study is its very restrictive selection criteria that allowed the comparison of two groups of individuals with no overlap in CD4 cell increments that could result in confounding results. Standardised protocols for the quantification of miRs are needed for cross-comparison to enhance their potential as biomarkers. Further studies will be required to determine any mechanistic relationship of miRs to diagnostic characteristics used to identify HIV-1 infected individuals at risk of poor cell recovery after suppressive ART.

Our study provides new evidence on the relationship between miRs and HIV-1 infection that could help improve the management of the individuals at HIV-1 diagnosis (at ART onset). The candidate miRs and cytokines signature sets presented here provide a starting point on which to establish definitive predictive and diagnostic signatures in the important clinical issue of the CD4 cell recovery after antiretroviral treatment. Besides, this signature must also be confirmed in an independent cohort. Also, since exosomes can have different origins, future studies will need to characterise the origin and nature of the HIV-1-associated miRs identified in this study.

## Supplementary information

Supplementary Dataset 1.

## Data Availability

The data measured and analysed for the current study are available from the corresponding author upon request.
