# Supplementary material for: New signatures of poor CD4 cell recovery after suppressive antiretroviral therapy in HIV-1-infected individuals: involvement of miR-192, IL-6, sCD14 and miR-144
Source: Sci Rep. 2020 Feb 19;10:2937. doi: 10.1038/s41598-020-60073-8 (PMC7031287; doi:10.1038/s41598-020-60073-8)
Supplement: Supplementary file 1 — Supplementary Dataset 1. [file 41598_2020_60073_MOESM1_ESM.docx]

**New** **signatures of poor CD4 cell recovery after suppressive antiretroviral therapy in HIV-1-infected individuals: involvement of miR-192, IL-6, sCD14 and miR-144**

Francisco Hernández-Walias ^a^, María Jesús Ruiz de León ^a^, Isaac Rosado-Sánchez ^b^, Esther Vázquez ^a^, Manuel Leal ^b,c^, Santiago Moreno ^a^, Francesc Vidal ^d^, Julià Blanco ^e,f^, Yolanda M. Pacheco ^b^, Alejandro Vallejo ^a^

FHW and MJRL have contributed equally to this work

a. Laboratory of Immunovirology, Department of Infectious Diseases. Health Research Institute Ramon y Cajal (IRyCIS), University Hospital Ramon y Cajal, Madrid, Spain.

b. Biomedicine Institute of Seville (IBiS), University Hospital Virgen del Rocío, Seville, Spain.

c. Department of Internal Medicine and Infectious Diseases. Hospital Viamed, Santa Ángela de la Cruz, Seville, Spain.

d. Infectious Diseases Unit and HIV/AIDS, Department of Internal Medicine, University Hospital Joan XXIII, IISPV, University Rovira i Virgili, Tarragona, Catalonia, Spain.

e. AIDS Research Institute IrsiCaixa-HIVACAT, Research Institute of Health Sciences Germans Trias i Pujol, Badalona, Catalonia, Spain.

f. University of Vic-University Central of Catalonia (UVIC-UCC), Vic, Catalonia, Spain

**Supplementary table S1.** miRs and cytokines levels regarding gender and HCV infection.

|  | **Optimal CD4 T cell recovery patients** | | | | | | |
| --- | --- | --- | --- | --- | --- | --- | --- |
|  | **Gender** | | |  | **HCV infection** | | |
|  | Males  N=46 | Females  N=8 | p |  | HCV infected patients  N=12 | HCV negative patients  N=20 | p |
| **A. miRs** |  |  |  |  |  |  |  |
| miR-106a  miR-140  miR-144  miR-221  miR-223  miR-320a  miR-409  miR-192  miR-24 | -0.17 [-0.83-1.36]  -0.37 [-1.35-0.64]  -3.15 [-4.48- -0.89]  -0.61 [-1.61- 0.52]  1.84 [3.01-5.03]  1.47 [0.42-3.03]  -3.42 [-4.66- -0.22]  -1.55 [-2.01- -0.07]  2.03 [0.24-3.83] | 0.22 [-1.62-1.69]  -0.03 [-1.20-0.62]  -4.52 [-4.83- -2.58]  -1.13 [-2.05-0.18]  1.29 [0.24-3.30]  2.13 [1.17-2.66]  -4.08 [-5.23- -3.43]  -0.26 [-0.86-1.03]  1.53 [-1.84-2.39] | 0.836  0.711  0.180  0.510  0.073  0.465  0.101  0.065  0.136 |  | 0.55 [-1.14-1.76]  -0.03 [-1.02-1.03]  -4.17 [-4.64- -1.98]  -1.12 [-1.45-0.67]  1.84 [0.41-3.37]  2.13 [1.29-3.06]  2.13 [1.29-3.06]  -4.10 [-4.74- -3.32]  -0.63 [-1.56-1.03] | -0.39 [-1.71-1.10]  -0.19 [-1.36-0.72]  -4.42 [-5.16- -3.62]  -1.44 [-1.99- -0.08]  2.09 [0.70-3.18]  1.45 [0.67-2.20]  -4.61 [-5.14- -3.91]  -1.64 [-2.01- -0.93]  1.93 [-0.63-3.21] | 0.272  0.366  0.182  0.552  0.984  0.107  0.125  **0.010**  0.985 |
| **B. Cytokines** |  |  |  |  |  |  |  |
| IL-2 (pg/ml)  IL-6 (ng/mL)  IL-17A (pg/mL)  TNF-α (pg/mL)  sCD14 (µg/ml)  ICAM (log_10_ ng/mL)  VCAM (log_10_ ng/mL) | 0.96 [0.64-1.34]  5.06 [3.19-6.78]  0.79 [0.58-1.15]  2.45 [1.43-4.43]  2.89 [1.75-3.40]  3.43 [2.27-4.18]  0.89 [0.61-1.16] | 1.14 [0.57-1.15]  3.05 [1.59-4.31]  0.72 [0.69-0.91]  3.52 [2.50-4.27]  2.98 [1.97-3.96]  3.15 [2.68-3.95]  1.01 [0.64-1.32] | 0.971  0.055  0.784  0.284  0.542  0.877  0.465 |  | 1.15 [0.64-1.29]  3.91 [2.79-8.50]  0.87 [0.68-1.11]  4.05 [1.84-5.22]  2.29 [1.56-3.04]  3.52 [2.93-3.97]  1.11 [0.86-1.32] | 1.23 [0.86-1.67]  4.35 [2.85-8.09]  0.88 [0.71-1.18]  2.38 [1.36-3.14]  2.80 [1.65-3.34]  2.79 [2.14-3.79]  0.88 [0.51-1.21] | 0.346  0.863  0.924  0.083  0.346  0.182  0.099 |

**Supplementary table S2**. ROC curve and likelihood ratio analysis of miR-192, IL-6 and sCD14 to predict poor CD4 T cell recovery, and miR-144 to diagnose poor CD4 T cell recovery.

|  | **ROC curve** | | | |  | **Likelihood ratio (LR)** | | | | |
| --- | --- | --- | --- | --- | --- | --- | --- | --- | --- | --- |
|  | AUC | p | Standard error | Range |  | Cut off | Sensitivity | Specificity | LR+ | LR- |
| **At cART onset** |  |  |  |  |  |  |  |  |  |  |
| miR-192 | 0.725 | 0.001 | 0.061 | 0.605-0.845 |  | 0.050 | 68% | 77.6% | 3.03 | 0.41 |
| IL6 | 0.760 | <0.001 | 0.056 | 0.650-0.870 |  | 6.475 | 68% | 75.9% | 2.82 | 0.42 |
| sCD14 | 0.640 | 0.046 | 0.065 | 0.514-0.767 |  | 3.450 | 44% | 77.8% | 1.98 | 0.72 |
| Combined: miR-192 + IL6 + sCD14 | 0.841 | <0.001 | 0.045 | 0.724-0.918 |  | 0.692 | 75.9% | 76% | 3.16 | 0.32 |
|  |  |  |  |  |  |  |  |  |  |  |
| **After 96 weeks under ART** |  |  |  |  |  |  |  |  |  |  |
| miR-144 | 0.730 | 0.008 | 0.077 | 0.579-0.882 |  | -2.801 | 75% | 71.4% | 2.63 | 0.35 |

**Supplementary table S3:** Immunovirologic characteristics of the patients after 96 weeks of cART.

|  | **Poor CD4 T cell recovery patients**  N=21 | **Optimal CD4 T cell recovery patients**  N=24 | p |
| --- | --- | --- | --- |
| CD4 T cell count (cells/mm3)  CD8 T cell count (cells/mm3)  CD4/CD8 ratio  CD4 count increment at 96 weeks | 206 [153-216]  868 [455-1207]  0.22 [0.16-0.42]  91 [62-134]^1^ | 433 [371-586]  912 [658-1342]  0.51 [0.38-0.87]  348 [284-407]^2^ | **<0.001**  0.403  **0.002**  **<0.001** |

Median and interquartile range (IQ_25-75_). Mann Whitney U test. Significant when p<0.05 in bold.
